# Household Livelihood Vulnerability to Climate Change in West China

**DOI:** 10.3390/ijerph19010551

**Published:** 2022-01-04

**Authors:** Jinyu Shen, Wei Duan, Yuqi Wang, Yijing Zhang

**Affiliations:** 1 School of Economics and Management, South China Agricultural University, Guangzhou 510642, China; shenjinyu@scau.edu.cn (J.S.); duanwei@scau.edu.cn (W.D.); 201823220113@stu.scau.edu.cn (Y.W.); 2 Research Center for Green Development of Agriculture, South China Agricultural University, Guangzhou 510642, China

**Keywords:** climate change, vulnerability, household livelihood vulnerability index, Western China

## Abstract

Climate change disproportionately affects natural resource-dependent communities in the ecologically vulnerable regions of western China. This study used the household livelihood vulnerability index under the Intergovernmental Panel on Climate Change (HLV-IPCC) to assess vulnerability. Data were collected from 823 households in Ningxia, Gansu, Guangxi, and Yunnan provinces, these being ecologically vulnerable regions in China. With a composite HLVI-IPCC and multiple regression model, the factors that affect households’ adaptive capability to HLVI-IPCC was estimated. Results indicate that Ningxia is the most vulnerable community, while Guangxi is the least vulnerable community across all indices. Moreover, Gansu has the heaviest sensitivity and exposure to climate change, whereas Ningxia has the highest adaptive capability to climate change. In addition, the age of household head and distance of the home to the town center had significant negative impacts on households’ adaptive capacity to HLVI-IPCC. The results also suggest that the HLVI assessment can provide an effective tool for local authorities to formulate prioritizing strategies with promoting climate-resilient development and increasing long-term adaptive capacity.

## 1. Introduction

Climate change disproportionately affects the resource-reliant poor, young, elderly, sick, and otherwise marginalized populations [1]. In addition, global climate change has a potential impact on human health, food production, water resources, human health and sustainable development of people around the world [2]. Therefore, the Department for International Development (DFID) has declared a focus on preventing adverse climate change effects and alleviating poverty in developing countries, especially in the ecologically vulnerable areas [3]. Specifically, some vulnerable ecological communities are highly dependent on climate-sensitive natural resources, and the impact on ecosystem services would be more significant in these communities. Households in these communities have limited adaptive capacity regarding the assets available to prepare for or respond to these climatic events [4]. Therefore, it is important to understand how households’ responses to climate change for achieving the goals of sustainable development are related to the environment [5,6].

To understand the impact of environmental change impact particular areas and the potential obstacles to effective responses, household livelihood vulnerability is part of a broader effort by scientists and policymakers. Several approaches for measuring vulnerability have developed widely across disciplines. For instance, the sustainable livelihoods framework (SLF), including “capital assets”—financial, human, social, physical, and natural—was not only used to understand poverty but can also assess exposure to natural disasters and civil conflict. However, climate change adds complexity to assessing household livelihood vulnerability accurately. Assessing vulnerability has been an essential step toward mitigating hazards and adapting to climate change [7]. Therefore, context-specific assessment methods were required to measure vulnerability [8]. The definitions and assessments of climate change vulnerability are inconsistent, as vulnerability is a notoriously difficult concept to standardize across disciplines [9]. According to the sixth assessment report of the Intergovernmental Panel on Climate Change (IPCC), vulnerability to climate change was defined as the propensity or predisposition to be adversely affected, which is involved sensitivity or susceptibility to harm and lack of capacity to cope and adapt [1]. Also, it is demonstrated as a hierarchical aggregation of three systems: physical, adverse exposure; sensitivity resulting from shock; and adaptive coping capacity to the negative impacts [10,11].

Numerous studies approach climate change vulnerability assessment by combining biophysical and social vulnerability using individual characteristics of households in specific communities [12,13]. Scholars have developed relevant assessment vulnerability indices to measure at the national [14], community [15], and household levels [16,17]. Furthermore, different indices with equally weighted indicators for each criterion have been developed, such as Climate Vulnerability [18], Livelihood Vulnerability Index (LVI) [19], Livelihood Effect Index (LEI) [20], and Multidimensional Livelihood Vulnerability Index (MLVI) [9]. However, some scholars argued that a composite vulnerability index with equal weight might not reveal the actual vulnerability, especially at the household level [21]. Therefore, given the inadequacy of such an assumption in most real-world contexts, some approaches instead of equal weights for indicators could be attempted [22,23]. Indexes such as the Social Vulnerability Index (SoVI) [24] and the Social Environment Vulnerability Index [25] were developed by using principal component analysis for weight indicators. According to the vulnerability assessment outcome, it is a micro-classify population to differentiate populations who may be more or less vulnerable and how they are vulnerable. From these results, the vulnerability index as a heuristic tool can identify households more likely to be vulnerable to future climate change to more effectively targetted with adaptation policies and also develop insight into the cause and structure of their vulnerability [26,27]. After this, households can better respond to climate problems, such as increasing temperatures or extreme weather events [28,29].

As one of the fastest developing countries, China is dominated by the East Asian monsoon cycle, and topography is highly vulnerable to climate change. In addition, China has suffered from climatic disasters throughout its history and continues to be vulnerable to climate change [30]. Consequently, numerous studies on the impacts of climate change on natural ecosystems in China have been conducted from the view of vulnerability assessment and the impacts on natural resources [31,32] and the socioeconomic adaptations of households [33,34]. However, it is unclear how climate change impacts household livelihoods and how poor people respond to climate change in the ecologically vulnerable regions of western China. Human activities and natural resources have been embedded in a complex social-ecological system, and integrate ecological, social, and economic values [35,36]. 

The study aims to assess the Household Livelihood Vulnerability Index under IPCC. With local perceptions and experiences of climate extremes, the vulnerability assessment index can help households respond, recover, and adapt to climate change better in ecologically vulnerable regions.

## 2. Study Methodology

### 2.1. Study Areas

China is a vast territory with varied topography and climate regimes and diverse ecosystems and high population pressure that has resulted in long-term human disturbances. Region location is a significant factor impacting ecological vulnerability [37]. The country encompasses various climate regimes from northern boreal to southern tropical and western arid to eastern and southern humid climate zones [38]. The areas examined in this study are located in the western region of China. Ningxia and Gansu are affected by their typical arid and semi-arid climates, while Yunnan and Guangxi are located in the mountainous regions. The geographical characteristics of study areas are shown in Table 1. 

**Figure 1:**
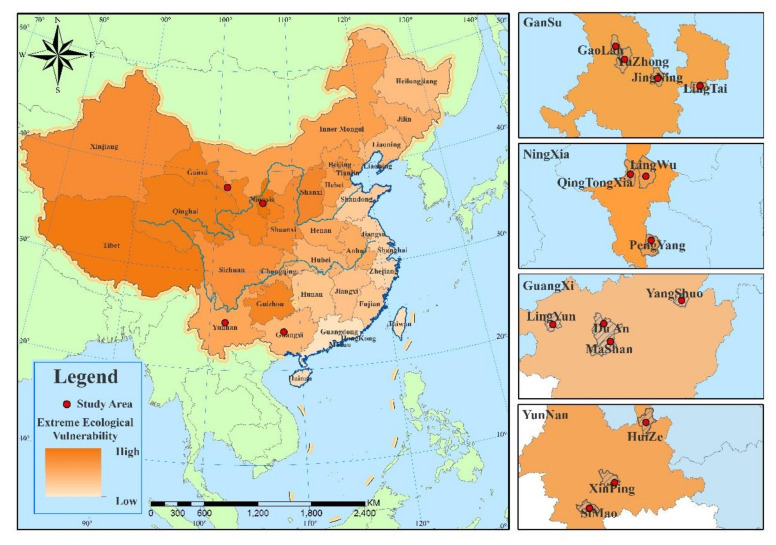
The vulnerable eco-distribution in China and the sites used in this study.

These regions are extremely sensitive to climate change and have been negatively affected due to their vulnerable ecologic environments, inferior educational systems, and crumbling infrastructure [39]. They also suffer from adverse effects such as food and water shortages and health deterioration problems. Overall, the adaptive capability of these study sites to climate change is much lower than eastern coastal developed regions in China. According to the National Program of Ecological Vulnerability Regions Protection [40], the vulnerable eco-distribution in China involved the study sites is shown in Figure 1.

### 2.2. Data Collection and Sampling

The sampling method of the household survey used was a combination of random sampling at the county level and stratified sampling at the village level. Specifically, four counties in each province were selected, and five to six villages were selected in each county according to households’ annual per capita income at high, middle, and low levels. About ten households were selected in each village. A survey of 823 households was investigated. Excluding the 31 invalid samples, the final valid sample count was 792 households, and interviews were primarily conducted face-to-face in isolation to secure the accurate information of the households. The survey collected data on each household’s social demographic profile, livelihood strategies, social networks, health, food, water, natural disasters, and climate variability. Additional indicators were added to the original LVI model because they were more suitable for local vulnerability to climate change. In the specific context of smallholder farm households in China, the diversity of agriculture, forests, and income have been strongly associated with a household’s capacity to manage environmental risk [41,42]. Hence, we incorporated forest diversity and other indicators and removed other indicators that were deemed non-relevant by local stakeholders. Table 2 summarizes the details used to collect the data for this study. In addition, combined with the SLF and the Hahn et al. [43] LVI, additional key-information interviews were conducted with a panel of local stakeholders, climate vulnerability experts, and researchers who study areas to modify and refine the indicators within the context of these specific regions.

Table 3 shows the socioeconomic characteristics of the households in the sample. Their average age is 53.78 years, and they have an average of 6.82 years of education. On average, each household comprises 4.3 persons, and 3.23 family members do non-farm work. The average total income of the households is about 47,403 RMB. Average cropland and forestland are 3.67 and 41.7 mu, respectively. In addition, the average distance of their home to the town center is 9.5 km. The descriptive statistics of the variables are shown in Table 3.

### 2.3. Household Livelihood Vulnerability Index-IPCC

**Figure 2:**
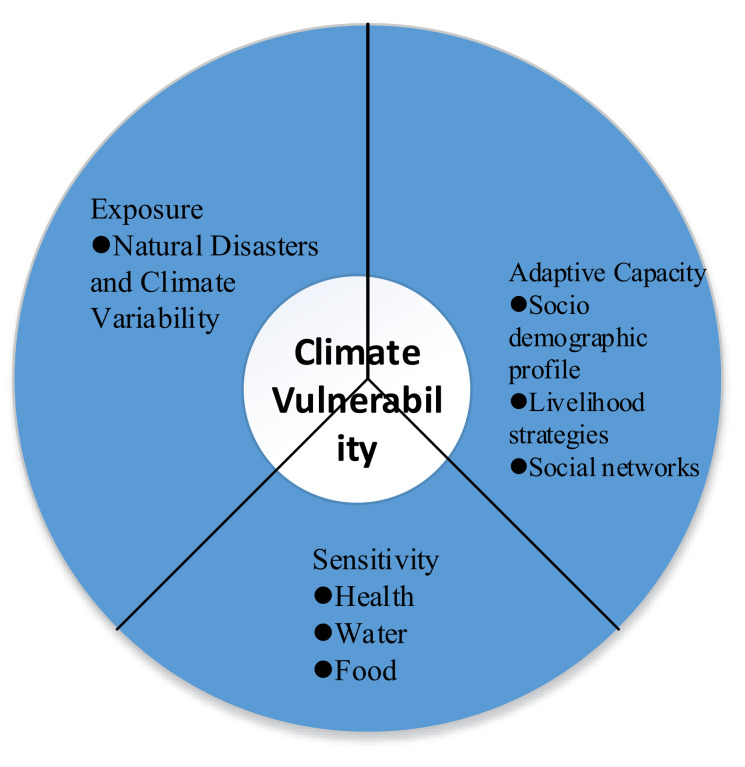
Grouping of the seven main indicators into the HLVI-IPCC.

Distinguished from previous studies, which are divided into five domains—demographic, social, economic, physical, and exposure to natural hazards—to assess the vulnerability at the household level, this study combined previous methods to construct a Household Livelihood Vulnerability Index (HLVI-IPCC), to estimate the impacts of climate change on communities in western China, such as Ningxia, Gansu, Guangxi, and Yunnan. The index-based vulnerability measurement included seven main indicators: Socio-demographic profile (SDP), livelihood strategies (LS), social networks (SN), health (H), food (F), water (W), and natural disasters and climate variability (NDCV). These criteria were developed based on a review of the literature on each main indicator [3,43] and the practicality of collecting the needed data through household surveys. Moreover, vulnerability to climate change was defined as a function of a system’s exposure and sensitivity to climate change as well as its capacity to adapt to the adverse effects. It does not provide a clear definition of these attributes or their relationships [44]. We conducted the HLVI under the IPCC framework, which entailed grouping the seven main indicators into the three categories shown in Figure 2.

#### 2.3.1. Calculating the Household Livelihood Vulnerability Index

Several indicators were combined to form the seven sub-indicators (Table 2). Once the appropriate indicators of exposure, sensitivity, and adaptive capacity had been identified from the literature, the structure for HLVI was developed as a three-level hierarchical structure. The AHP, proposed by Saaty [45], is a popular and widely used technique for multi-criteria decision-making. It determines the relative weights of the decision criteria and the relative priorities of the indicators and helps decision-makers come to conclusions that best suit their goals and understanding. AHP employs the subjective values and preferences of the decision-makers, including three professors in ecological conversation, three forestry managers, three agricultural managers, three climate change managers, and three environmental managers, while utilizing their varying levels of capabilities, expert knowledge, and experiences to bring out a quantitative result that is usable in strategic evaluations.

We first organized the exposure, sensitivity, and adaptive capacity indices into a three-level hierarchy. The top-level corresponded to the overall goal of the analysis (i.e., classify households’ vulnerability to climate change) to obtain the respective indicator weights associated with the concepts of the main indicators. The second level corresponds to the set criteria to specify the overall goal, and the lowest level represents the alternatives being evaluated.

#### 2.3.2. Transformation of the Raw Data into Commensurate Indicator Values

The variables in this study contained incommensurate data (e.g., in different unions and percentages, ratios, and indices), so indicators were necessary to unify all data into a standard 0–1 scale with appropriate measurement ratio properties.

(1) $$\mathrm{Index}Y_{sd}=\frac{Y_{j}-Y_{min}}{Y_{max}-Y_{min}}$$

where $\mathrm{Index}\;Y_{sd}$ is the standardized index for $Y_{j}$, $Y_{j}$ (the original indicator for district *j*), and $Y_{min}$ and $Y_{max}$ are the minimum and maximum values, respectively, for each sub-indicator determined by using data from the communities. These minimum and maximum values were used to transform each indicator into a standardized index.

Once the indicator Index $Y_{sd}$ was obtained and the weight $W_{i}$ and $W_{j}$ were also assumed for each contributing indicator through AHP, we proceeded with the HLVI based on the following equation:

(2) $$\mathrm{HLVI}=\overset{m}{\underset{j=1}{\sum }}W_{j}(\overset{n}{\underset{i=1}{\sum }}Y_{sd}W_{i})$$

where $W_{i}$ is the weight of sub-indicator *i*, and *i* = 1, 2,…, *n*; $W_{i}$ is the weight of main indicator and *j* = 1, 2,…, *n*.

#### 2.3.3. Calculating the Household Livelihood Vulnerability Index under IPCC (HLVI-IPCC)

The three factors contributing to HLVI-IPCC is calculated based on the following equation, respectively:

(3) $$CF_{j}=\frac{\sum_{i=1}^{n}w_{Mi}M_{ji}}{\sum_{i=1}^{n}w_{Mi}}$$

where *CFj* is an IPCC-defined contributing factor (exposure, sensitivity, or adaptive capacity) for community *j*, *Mji* are the main indicators for community *j* indexed by *i*, *W_Mi_* is the weight of each main indicator, and *n* is the number of main indicators in each contributing factor. Once exposure, sensitivity, and adaptive capacity were calculated, the three contributing factors were combined using the following equation:

(4) $$\mathrm{HLVI}-{\mathrm{IPCC}}_{j}=\left(e_{j}-\alpha_{j}\right){\;*\;S}_{j}$$

where HLVI–IPCC*j* is the HLVI for community *j* expressed using the IPCC vulnerability framework, *e* is the calculated exposure score for community *j* (representing the natural disasters and climate variability main indicators), α is the calculated adaptive capacity score for community *j* (weighted average of the socio-demographic, livelihood strategies, and social networks main indicators), and *S* is the calculated sensitivity score for community *j* (weighted average of the heath, food, and water main indicators). The HLVI–IPCC was scaled from −1 (least vulnerable) to +1 (most vulnerable).

## 3. Results and Discussion

### 3.1. Overall Household Livelihood Vulnerability Index

The results of the data analysis of HLVI for the study communities are reported in Table 4, which shows the actual value (AV) and standard value (sd) of each indicator, as well as the minimum and maximum values by respondent households. Results are based on 28 weighted sub-indicators individual contributions to the pairwise comparison matrix. Furthermore, the seven main indicators were calculated and then assessed via the HLVI with Equation (4). Data suggest that Ningxia was the most vulnerable to climate change among the communities, with an HLVI of 0.449 compared to Gansu, at 0.439; Yunnan, at 0.37; and Guangxi, at 0.36.

**Figure 3:**
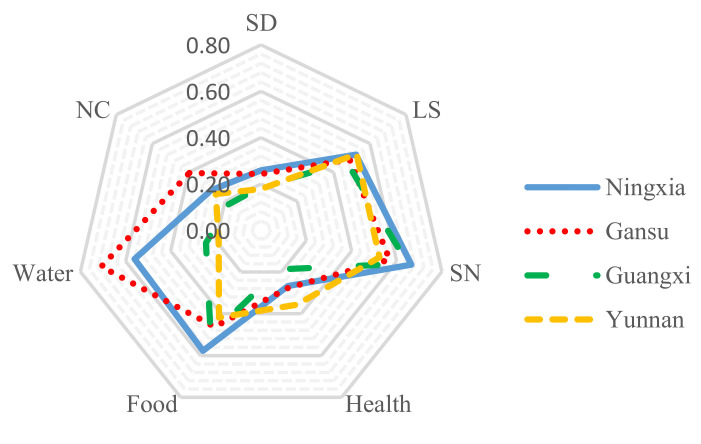
Vulnerability spider diagram of the main indicators of the Household Livelihood Vulnerability Index (HLVI) for Ningxia, Gansu, Guangxi, and Yunnan, China.

The results of the main indicator calculations are also presented collectively in a spider diagram (Figure 3). The scale of the diagram ranges from 0 (less vulnerable) at the center of the web, increasing to 0.8 (most vulnerable) at the outside edge, in 0.2 unit increments. The results suggest that Gansu had the most Water vulnerability (0.707); with natural disasters and climate change (0.395); Yunnan was the most vulnerable in terms of LS (0.526) with H (0.356); However, Ningxia was the most vulnerable community in those dimensions and Guangxi was the least vulnerable community across all dimensions.

### 3.2. The Household Livelihood Vulnerability Index under IPCC

The results of HLVI-IPCC indicated in Table 5 suggested slightly different degrees of vulnerability among the four communities on a scale from −1 to +1, as recommended by Hahn (2009) and Shah et al. (2013). In comparing adaptive capability scores, Ningxia has a slightly higher value (0.471) than Gansu (0.427), Guangxi (0.422), and Yunnan (0.410); meanwhile, exposure scores of all four communities shows Gansu (0.0015) with a higher score than Ningxia (0.0011), Yunnan (0.01), and Guangxi (0.0007). Regarding sensitivity to climate change, Ningxia (0.487) and Guangxi (0.5) had higher scores than those of Guangxi (0.322) and Yunnan (0.317). Overall, Yunnan’s HVLI-IPCC score of −0.127 indicated that it was the most vulnerable to climate change, followed by Guangxi (−0.134), Gansu (−0.206), and Ningxia (−0.224).

#### 3.2.1. Adaptive Capability to Climate Change

As shown in Table 5, the proportion of households with members who travel to other communities to work was highest in Ningxia. This is mainly due to rural labor migration into cities in China by household members who believe they can obtain a greater income level by working outside of their local agricultural and forestry sectors. However, the income reported by these household members was not significantly greater than income reported by those working in the local agricultural and forestry sectors in Gansu and Yunnan. In Gansu, household members do not travel far to work, as the area has an undeveloped economy with consistently low wages. On the other hand, in Yunnan, household members typically engage more frequently in agricultural and forestry activities to derive income. Furthermore, although the geographical locations of Ningxia and Gansu and are not much more disadvantageous than the locations of Guangxi and Yunnan, Ningxia and Gansu households reported more children, elderly members, and female heads of household with low levels of education.

In terms of the degree that households are dependent on relatives and friends for financial assistance and other help, we assumed that a household that receives money or in-kind assistance often but offers little assistance to others is more insecure and vulnerable compared to those that give their money and time to help others. The findings in this study showed that the southwestern provinces leaned higher toward borrowing than in the northwestern provinces. Many households in Yunnan reported they did not participate in local activities to receive help from the government. It is possible that the local preferential policy of households was not effective and that households had little knowledge about the preferential policy.

Furthermore, to explore the determinants of households’ adaptive capacity to HLVI-IPCC, the multiple regression model was used to estimate. As is shown in the Table 6, the age of the household head and distance of home to the town center had significant negative impacts on households’ adaptive capacity to HLVI-IPCC. The educational level of the household head, non-farm employment and total income of the household had significant positive impact on households’ adaptive capacity to HLVI-IPCC.

#### 3.2.2. Sensitivity to Climate Change

Despite Yunnan households reporting a shorter average time to travel to a health facility compared to the other communities, households in this province reported a higher proportion of members who suffer from chronic illnesses and have missed work in the last two weeks due to illness. Hence, the opportunity for household members to work outside the community was decreased. The local spread of many climate-sensitive diseases as a result of climate change has affected China (Bi); therefore, more consideration should be paid to the prevention and control of chronic diseases in the future. Strengthening basic health infrastructure in Ningxia and Gansu in an attempt to reduce the travel time between households and a health facility should also be considered to reduce this local health vulnerability.

In general, climate change increases the instability of the agricultural industry and the volatility of crop yields in China. Even worse, it can result in the overall failure of agricultural productivity if the government does not take any measures to relieve the trend [46]. Ningxia and Gansu, located in northwestern China, were more significantly affected and sensitive to arid climate change. Water resource demand is greater in these provinces due to the warm climate, which increases production costs and investment towards becoming more adaptive to climate vulnerability. Although climate change has been shown to alter productivity in China, it is not always negative [47]; for example, when hundreds of cattle deaths and hectares of crops reduced by climate change threatened the lives of households in Ningxia, more than 90% reported that they then began relying on their own farms for food. Households also reported that crop diversity was determined by the local ecologic environment. In contrast to the droughts of Ningxia, Guangxi is flood-prone. The households that showed a higher proportion of living on their own farm food with a greater variety of crops revealed that they did not trade or store crops. It was found that the amount of serious flooding and waterlogging in Guangxi that has occurred in the past 10 years is much higher than 30 years ago, which at the time induced great damage to crops and caused economic losses to households [48]. This indicates that it is important to highlight the implications for reducing food sensitivity to climate change. The development of water-saving agriculture, the protecting and improving of the ecological environment, and improving the adaptability of agriculture in arid climates is recommended in the northwest part of China, whereas strengthening debris and landslide prediction and implementing soil and water conservation projects is recommended in the southwestern regions. 

Agriculture activities were based on water resources, particularly during drought years. The decrease of runoff has a negative impact on agriculture, even if there is no direct impact on crop yield. The northwestern part of China is more drought-prone, so the Ningxia and Gansu households have a relatively higher proportion of a lack of pipe-borne water and regular water supply than households in Guangxi and Yunnan. Most Guangxi and Yunnan households had to utilize a natural water system, spending more days storing water. During the drought that occurred in the winter of 2013, 643,000 households did not have enough drinking water resources. Insufficient water for agriculture is therefore expected through the 2020s and 2040s due to the increases in water demand for non-agricultural uses, although precipitation may increase in some areas of China [48]. Recently, the establishment of freshwater pumping stations and increasing irrigation equipment have been supported by local Ningxia and Gansu governments. These strategies can mitigate water vulnerability impact on agricultural and forestry activities, though a consultation mechanism should also be established between upstream and downstream channels regarding water resource allocation and water management, as recommended by Ma et al. [49]. Reservoirs for river discharge and irrigation water supply should be developed in northwestern China.

#### 3.2.3. Exposure to Climate Change

Gansu households reported a higher absolute number of natural disasters over the past 10 years, and the variability in the monthly average minimum and maximum daily temperature and precipitation was greater than in the other communities. Gansu’s highest exposure score to this vulnerability is attributed to a lack of information dissemination initiatives. Interviews revealed that households in hazard-prone communities did not want to risk losing assets such as cattle, poultry, and household belongings during catastrophes. None of the four study communities had established early warning systems to alert people in an event of natural disaster or climatic event; therefore, people had to rely on forecasting by the national climate warning system. Local early warning systems and community preparedness plans may help communities decrease the adverse impacts of extreme weather events. For the purposes of this study, we chose 10 years as a timeframe for obtaining indicators to reduce the likelihood of household members not remembering an accurate number of disasters that occurred further in the past. The time standard could be lengthened in future studies to better understand the complex characteristics of climate change.

## 4. Conclusions

Our study showed that Ningxia was the most vulnerable community in the HVLI with the highest vulnerability in terms of socio-demographics, social networks, and food. Additionally, Gansu was identified as having the greatest water vulnerability and natural disasters and climate variability; Yunnan was identified as being the most vulnerable community in terms of livelihood strategies such as heath, and Guangxi was the least vulnerable across all the indices. Overall, for the vulnerability indicators, Ningxia was the most vulnerable community to all three IPCC-defined contributing factors to climate change with the greatest adaptive capability, and Gansu had the most significant exposure to climate change. 

We also considered appropriate processes and tools required to assess HLVI. Through slight modification, a number of indicators were added or revised to better fit the context of the western regions of China; likewise, some indicators were dropped as being less relevant. This study provides two contributions to the development of our understanding of vulnerability indicators. First, it provides an HLVI-IPCC assessment through a multi criteria decision analysis in the weighting of indicators. Second, coping better with climate change is critical to increasing the long-term adaptive capacity of vulnerable household groups. Therefore, it is hoped that this HLVI assessment will provide an effective tool for local authorities to tailor policy to promote climate-resilient development, as well as to allow government to make more effective resource allocation decisions on improving infrastructure and funds.

## Figures and Tables

**Table 1:** The geographical characteristics of study areas.

	Ningxia	Gansu	Guangxi	Yunnan
Location	N(35°14′–39°23′) E(104°17′–107°39′)	N(32°31′–42°57′) E(92°13′–108°46′)	N(20°54′–26°24′) E(104°26′–112°04′)	N(26°57′–27°12′) E(114°17′–114°97′)
Climate	Temperate continental climate in the northern part, with a subtropical monsoon climate in the southern part	Subtropical monsoon climate	Temperate continental climate	Subtropical monsoon climate in the northern part, with a tropical monsoon climate in the southern part
Temperature	Average annual temperature around 10.3 °C	Average annual temperature range from 16.5 °C to 23.1 °C	Average annual temperature range from 5 °C to 9 °C	Average annual temperature around 15 °C
Rainfall	Average annual rainfall 450 mm	Rainfall range between 1500 mm and 2000 mm	Rainfall range between 300 mm and 500 mm	Rainfall range between 1100 mm and 1600 mm
Land use pattern	Cropland area: 1195.4 (1000 Ha) Forestland area: 952.6 (1000 Ha)	Cropland area: 5209.5 (1000 Ha) Forestland area: 7962.8 (1000 Ha)	Cropland area: 3307.6 (1000 Ha) Forestland area: 16,095.2 (1000 Ha)	Cropland area: 5395.5 (1000 Ha) Forestland area: 24,969 (1000 Ha)

**Table 2:** Main indicators and sub-indicators comprising the Household Livelihood Vulnerability Index (HLVI) developed for the study.

Main-indicators	Sub-Indicators	Measurement
Socio-Demographic Profile	Dependency ratio (SD_1_)	$\frac{\mathrm{The}\;\mathrm{number}\;\mathrm{of}\;\mathrm{households}\;\left(\langle 15\mathrm{years}\;+\rangle 65\;\mathrm{years}\right)}{\mathrm{The}\;\mathrm{number}\;\mathrm{of}\;\mathrm{households}\;\left(\mathrm{betwwen}19\;\mathrm{and}\;65\;\mathrm{years}\;\right)}$
	Percentage of female-headed households (SD_2_)	$\frac{\mathrm{The}\;\mathrm{number}\;\mathrm{of}\;\mathrm{female}\;\mathrm{headed}\;\mathrm{households}\;}{\mathrm{The}\;\mathrm{number}\;\mathrm{of}\;\left(\mathrm{male}\;\mathrm{and}\;\mathrm{female}-\mathrm{headed}\;\mathrm{households}\right)}$
	Avg. of the educational level of headed households (SD_3_)	Average of the educational level of head households
	Percentage of the household head has not finished primary school (SD_4_)	$\frac{\mathrm{The}\;\mathrm{numberrof}\;\mathrm{headed}\;\mathrm{households}\;\mathrm{not}\;\mathrm{fnished}\;\mathrm{primary}\;\mathrm{school}\;}{\mathrm{The}\;\mathrm{number}\;\mathrm{of}\;\mathrm{all}\;\mathrm{households}}$
Livelihood Strategies	Percentage of households engaging in off-farm work outside the community (LS_1_)	$\frac{\mathrm{Members}\;\mathrm{working}\;\mathrm{outside}\;\mathrm{the}\;\mathrm{community}\;}{\mathrm{The}\;\mathrm{number}\;\mathrm{of}\;\mathrm{all}\;\mathrm{households}}$
	Percentage of households depends on agriculture/forest(LS_2_)	$\frac{\mathrm{The}\;\mathrm{number}\;\mathrm{of}\;\mathrm{household}\;\mathrm{main}\;\mathrm{income}\;\mathrm{source}\;\mathrm{is}\;\mathrm{agriculture}/\mathrm{forest}\;}{\mathrm{The}\;\mathrm{number}\;\mathrm{of}\;\mathrm{all}\;\mathrm{households}}$
	Percentage of households without non-agriculture and non-forest livelihood income contribution (LS_3_)	Percentage of households reporting livelihoods other than agriculture/forest as the main source of income
	Avg. agricultural livelihood diversity index (LS_4_)	The inverse of (the number of agricultural livelihood activities + 1)
	Avg. forestry livelihood diversity index (LS_5_)	Same as above
Social Networks	Percentage of households internet users in household without using internet (SN_1_)	$\frac{\mathrm{The}\;\mathrm{number}\;\mathrm{of}\;\mathrm{household}\;\mathrm{use}\;\mathrm{internet}+1\;}{\mathrm{The}\;\mathrm{number}\;\mathrm{of}\;\mathrm{household}\;\mathrm{without}\;\mathrm{using}\;\mathrm{internet}+1}$
	Avg. borrow: lend ratio (SN_2_)	Ratio of a household borrowing money in the past month to a household lending money in the past month
	Percentage of households have participated in village activities for help in last year (SN_3_)	Percentage of households that reported that they have participated in village activities in last year
Health	Avg. time to clinic/hospital (H_1_)	Average time to go to the nearest clinic/hospital
	Percentage of households with members suffering chronic illness/severe illness (H_2_)	Percentage of households reporting at least one member with chronic disease or severe illness
	Percentage of medical expenses for the sick member (H_3_)	Percentage of households medical expenses in their total expenses
Food	Percentage of households primarily dependent on self-farmed food (F_1_)	Percentage of households that get their food primarily from their land
	Avg. crop diversity index (F_2_)	the inverse of (the number of crops grown by household +1)
	Percentage of households that do not sell/barter crops for other food supplies (F_3_)	Percentage of households unable to trade self-grown crops
	Percentage of households that do not save crops (F_4_)	Percentage of households buy their food always without planting crops
Water	Percentage of household without piped water (W_1_)	Percentage of households not receiving water through the public water system
	Percentage of households utilizing natural water system (W_2_)	Percentage of households obtaining water from wells, rainwater, springs, and other means apart from the public system
	Avg. days without regular water supply per year (W_3_)	Percentage of households reporting that water is not available at their primary water supply
	Inverse of number of days with water supply from stored source in the house (W_4_)	Average water supply security per household
Natural disasters and climate variables	Avg. number of floods/droughts in past 3 years (ND_1_)	Total number of floods, droughts, reported by households in the past 3 years
	Avg. number of pests in past 3 years (ND_2_)	Total number of floods, droughts reported by households in the past 3 years
	Mean standard deviation of monthly avg.max.daily temperature in last 5 years (ND_3_)	Standard deviation of the average daily maximum temperature by month between 2001–2010 was averaged for each area
	Mean standard deviation of monthly avg.min.daily temperature in last 10 years (ND_4_)	Standard deviation of the average daily minimum temperature by month between 2001–2010 was averaged for each area
	Mean standard deviation of monthly avg. precipitation (ND_5_)	Standard deviation of the average monthly precipitation between 2000–2019 was averaged, or each area

Note: All indicators are based on Hahn et al. [42], and some modifications are based on the investigation of the study areas. The higher the value is, the higher the vulnerability.

**Table 3:** Descriptive statistics of selected variables.

Variables	Definition	Mean	SD
Age	Age of household head (years)	53.78	12.02
Education	Educational level of household head (years)	6.82	3.35
Healthy	Physical condition of household head (if sick = 1)	0.28	0.45
Households size	Number of family members	4.30	1.76
Non-farm employment	Number of family members with non-farm employment	3.23	1.52
Cropland area	Household farmland size (mu)	3.67	5.68
Forestland area	Household forestland size (mu)	41.70	61.53
Total income	Household income in RMB	47,403.4	38,125.44
Distance of home to the town center	Distance of home to the town center (km)	9.50	11.00

**Table 4:** Overall results of indicators in HLVI for Ningxia, Gansu, Guangxi, and Yunnan provinces.

Indicator	Units	Ningxia		Gansu		Guangxi		Yunnan		Max. Value	Min. Value	*W_i_*
		AV	*Y_sd_*	AV	*Y_sd_*	AV	*Y_sd_*	AV	*Y_sd_*			
SD_1_	Ratio	1.56	0.195	1.23	0.154	1.02	0.128	0.96	0.12	8	0	0.505
SD_2_	%	25.1	0.251	25.2	0.252	23.2	0.232	16.4	0.164	100	0	0.157
SD_3_	1/Years	0.022	0.403	0.021	0.351	0.02	0.338	0.023	0.435	0	0	0.093
SD_4_	%	34	0.34	38	0.38	23	0.23	21	0.21	100	0	0.245
LS_1_	%	81	0.81	75	0.75	72	0.72	70	0.7	100	0	0.34
LS_2_	%	45	0.45	45	0.45	33	0.33	56	0.56	100	0	0.258
LS_3_	%	46	0.46	44	0.44	55	0.55	31	0.31	100	0	0.096
LS_4_	1/No. of livelihoods	0.31	0.31	0.17	0.17	0.22	0.22	0.26	0.26	1	0	0.126
LS_5_	1/No. of livelihoods	0.27	0.27	0.3	0.3	0.34	0.34	0.45	0.45	1	0	0.18
SN_1_	Ratio	1.44	0.148	1.07	0.1	1.26	0.125	1.68	0.179	8	0	0.239
SN_2_	Ratio	1.03	0.353	1.06	0.373	1.54	0.693	1.32	0.547	2	1	0.137
SN_3_	%	93	0.93	79	0.79	84	0.84	65	0.65	100	0	0.625
H_1_	Min	165.9	0.038	278.5	0.064	89.4	0.02	94.7	0.022	4320	1	0.157
H_2_	%	34	0.34	37	0.37	25	0.25	46	0.46	100	0	0.594
H_3_	%	24	0.24	18	0.18	14	0.14	32	0.32	100	0	0.249
F_1_	%	96.4	0.964	65.5	0.655	92.3	0.923	73.2	0.732	100	0	0.456
F_2_	1/No. of crops	0.26	0.178	0.23	0.144	0.21	0.122	0.28	0.2	1	0	0.1
F_3_	%	26.5	0.265	32.3	0.323	43.2	0.432	25.1	0.251	100	0	0.122
F_4_	%	27.3	0.273	34.1	0.341	5.2	0.052	10.5	0.105	100	0	0.322
W_1_	%	64.5	0.645	84.3	0.843	97.4	0.974	90.2	0.902	100	0	0.086
W_2_	%	43	0.43	52	0.52	9.8	0.098	5.4	0.054	100	0	0.265
W_3_	Days	5.32	0.76	6.71	0.959	1.82	0.26	1.26	0.18	7	0	0.507
W_4_	1/Days	0.04	0.039	0.08	0.079	0.02	0.019	0.03	0.029	1	0	0.142
ND_1_	Count	5.4	0.45	7.8	0.65	4.6	0.383	3.2	0.267	12	0	0.218
ND_2_	Count	8.9	0.89	9.3	0.93	4.2	0.42	5.7	0.57	10	0	0.076
ND_3_	°C	0.7	0.2	0.9	0.333	0.5	0.067	1.3	0.6	2	0	0.116
ND_4_	°C	0.4	0.034	0.8	0.172	0.7	0.138	0.9	0.207	3	0	0.144
ND_5_	mm	52.6	0.187	73.2	0.268	25.7	0.08	35.7	0.12	259	5	0.447
HLVI		0.449		0.439		0.36		0.37				

**Table 5:** The weight and score for main indicators and HLVI-IPCC value for Ningxia, Gansu, Guangxi, and Yunnan.

Main Component	$W_{j}$	Ningxia	Gansu	Guangxi	Yunnan	IPCC Contributing Factor	Ningxia	Gansu	Guangxi	Yunnan
		M	M	M	M					
Socio-demographic profile	0.258	0.259	0.243	0.189	0.178	Adaptive capacity	0.471	0.427	0.422	0.410
Livelihood strategies	0.404	0.523	0.489	0.472	0.526					
Social networks	0.057	0.665	0.569	0.649	0.524					
Health	0.05	0.268	0.275	0.187	0.356	Sensitivity	0.487	0.500	0.322	0.317
Food	0.077	0.577	0.462	0.502	0.418					
Water	0.115	0.56	0.707	0.244	0.187					
Natural disasters and climate variable	0.038	0.277	0.395	0.179	0.254	Exposure	0.011	0.015	0.007	0.01

**Table 6:** Regression results of determinants of HLVI-IPCC.

	Dependent Variable: Adaptive Capacity to HLVI-IPCC	
	Coefficient	Std. Err
Age	−0.773 ***	0.130
Education	0.196 *	0.114
Health	0.059	0.046
Households size	0.022	0.164
Non-farm employment	0.625 **	0.204
Cropland area	−0.036	0.209
Forestland area	0.768	0.670
Total income(log)	1.670 ***	0.324
Distance of home to the town center	−0.317 **	0.161
Constant	1.006 ***	0.103
R^2^	0.501	

Note: ***, **, and * denote significance at the 1, 5, and 10% levels, respectively.

## Data Availability

Data available on request due to restrictions.
